# Distributed Caregiving for Cognitively Impaired Individuals: A Case Report

**DOI:** 10.7759/cureus.34677

**Published:** 2023-02-06

**Authors:** Yara Alemi, Blaise Loughman, Maria Uriyo

**Affiliations:** 1 Medicine, Royal College of Surgeons in Ireland, Dublin, IRL; 2 Health Administration and Policy, George Mason University, Fairfax, USA

**Keywords:** home platforms, cognitive impairment, case study, caregiver burden, care navigation

## Abstract

Many caregivers of people with cognitive impairment spend a significant amount of their time helping patients with instrumental daily functions. Distributed caregiving is an innovative model designed to reduce an individual caregiver’s time burden and increase the likelihood of continued independent living for the patient.

Echo Show and Google Home platforms were used to enable the participation of remote family members in caregiving, specifically the socialization and entertainment of a person with cognitive impairment. Caregiver interviews, review of medical records, and case study analysis were used to measure caregiver burden, after distributing some components of caregiving to distant family members with human-in-the-loop artificial intelligence.

This case explores the use of Alexa, Echo Show, and other commercial technologies in the management of a patient with cognitive impairment. The human-in-the-loop system introduced in this case study is a creative, accessible, low-cost, and sustainable way to potentially reduce caregiver burden and improve patient outcomes with targeted intervention. Targeted distributed caregiving reduced time spent in caregiving, reduced caregiver guilt and frustration, improved patient’s compliance with requests for behavior changes (e.g., voiding before leaving the house), and improved the relationship between the caregiver and the person with cognitive impairment.

This case study demonstrates how distributed caregiving, including human-in-the-loop artificial intelligence, can lead to better use of technology in reducing the social isolation of persons with cognitive impairment and in reducing caregiver burden.

## Introduction

The challenge, in this case, was to reduce the burden on the primary caregiver by engaging distant caregivers in a distributed model of care. Historically, distant caregivers can be engaged in financial management decisions, but local caregivers are engaged in all aspects of the life of the independent living cognitively impaired elderly.

In the United States, more than 2.3 million persons with cognitive impairments live independently but need regular help from family members or friends to carry out instrumental activities of daily living [1]. As many of these conditions are degenerative, the caregivers’ tasks scale with the patients’ decline and can include assistance with walking, shopping for food, preparation of meals, toileting, eating, coordination with the health care system, maintaining medication regimens, and addressing end‐of‐life issues and advance care decision-making [2]. Many caregivers experience a burden and can become overwhelmed by the intensive and repetitive nature of caregiving [2-6]. Caregiver burden is the main reason persons with cognitive impairment are prematurely sent to nursing homes [2,4,7-9]. The burden also has known negative effects on the caregiver [6,10]. Even small reductions in caregiver burden have the potential for massive impacts on the quality of life of both the patient and the caregiver and can help avoid or defer nursing home entry [2].

Many interventions have been proposed with the intent to help caregivers and reduce their burdens, with a variety of methods. Some seek to improve the health and well‐being of caregivers through providing peer support to caregivers or providing respite from caregiving [2,4,7-9,11-13]. Some attempt to use technology to make caregiving easier [14-19]. There are NIH-funded studies focused on evaluating the impact of technology on caregiving [20,21]. More recently, randomized trials have focused on the use of robots to reduce the social isolation of persons with cognitive impairments [22-26]. The State of New York is experimenting with ElliQ, a robot companion shown to enhance the health of elderly patients [27]. 

Human-in-the-loop Technology enables remote individuals to observe and interact with the person with cognitive impairment, as well as to take actions (e.g. turn smart switches on or off remotely). While not completely automated, these human-in-the-loop automation are flexible and have the potential to be trusted more than fully autonomous systems by elderly individuals.

Description of distributed caregiving

Distributed caregiving refers to the use of technology to enable family or friends who are not living with an elderly person to assist in carrying out instrumental daily functions. Remote caregivers are provided with technology that allows them to see the home, interact with the elderly person, and manage remote devices in the home. Distributed caregivers can do a variety of tasks, including medication intake observation, monitoring of cooking and prevention of fires in the kitchen, reminders for time-based voiding, shopping for food, calling for the repair of home systems, and so on. In this case study, we focus on the role of distributed caregiving in reducing social isolation.

## Case presentation

Study design

We conducted this study using qualitative interviews with the person with cognitive impairment, the local caregiver, and the distant caregiver. The caregivers were asked to estimate the time they spent on various activities. Both caregivers who consented to this study have had the opportunity to provide feedback on this report.

To enable the distant caregiver to interact with the home, we organized two home platforms (Echo Show and Google Home) next to each other. These are voice-controlled intelligent personal assistants. The Echo Show is an Amazon product. With the coding available in the appendix, Echo Show was used for communication with the person with cognitive impairment and for sending commands by the distant caregiver to the Google Home platform. The Google Home platform was used to (a) play music from earlier periods that were familiar to the person with cognitive impairment, (b) manage problems with turning the television on and off, and (c) set timers and reminders. At regular intervals, usually minutes before the departure of the local caregiver, the distant caregiver would call through the Echo Show. The system was set up with the consent of the person with cognitive impairment so that it did not require the person to answer the call, using a pre-existing feature called “dropping in”. In general, we have found that individuals with cognitive impairment have difficulty managing technology, except when it is voice activated [28]. The use of the drop-in feature allowed the person with cognitive impairment to avoid handling the Echo Show device to answer incoming calls, thus removing one step in the use of the technology.

Once the call occurred, the distant caregiver and the elderly person would have conversations on a variety of topics. To make sure that the conversation would continue for at least thirty minutes, several steps were employed. Traditionally, these phone calls are goal-oriented and therefore, the conversation was short. In contrast, the conversations between a distant caregiver and a person with cognitive impairment needed to be flexible and long. To accomplish longer conversations, the distant caregiver had a series of regular topics that were repeated day after day. These included:

(a) Reading poetry to and asking the elderly person to read to a distant caregiver

(b) Playing games such as 20 questions

(c) Calling other friends and family members

(d) Asking about the content of the last meal

(e) Keeping the person with cognitive impairment updated on broad issues related to her financial affairs.

Topics were chosen to engage the person with cognitive impairment in conversation, although sometimes responses were formulaic and repetitive. For example, calls to friends would start with salutations, then referencing old events, but would end soon as there was little to discuss. In contrast, reading poetry was a long and engaging activity.

Methods of training and implementation

To organize the distributed caregiving, we had to educate the distant family member on the use of the Echo Show, and the role of distant family members in selecting topics for conversation. We also had to increase his internet speed to allow for a stable connection. Internet service providers can sometimes reduce speed on calls in certain circumstances, which can result in portions of the spoken words not being transmitted, exacerbating the confusion in the mind of the person with cognitive impairment. It is important that the internet connection is a high speed and stability. It is also important to train the distant caregiver to remain flexible. The purpose of communication is not to accomplish any specific task but to keep up with the interests of the person with cognitive impairment. For this study, it is the process, and the length of the process, that matters and not any behavioral changes accomplished through these conversations.

The person with cognitive impairment was introduced to the system gradually by the local caregiver. First, the devices were used by the local caregiver to play music, while the local caregiver was on site. At this point, the person with cognitive impairment complained that the devices were not useful. The access to the music of her youth, however, was an important attraction for the home platform. Second, the Echo Show was used for communication with the distant caregiver with aid of the local caregiver. The person with cognitive impairment was able to quickly learn how to make calls using her voice commands. As the trial continued, the distant caregiver began to use the Echo Show to increase socialization, monitor activity levels, generate reminders, and turn devices such as television or music, on and off. The gradual introduction of home platforms and communication devices is necessary for success due to the patient potentially rejecting the device. For example, in a previous attempt with a different person, the person with cognitive impairment categorically refused the home platforms when these platforms were not introduced gradually. 

Family conflict in caring for elderly individuals is a constant issue [29]. It is important to train both the distant and local caregivers in reducing open conflict and in appreciating each party’s contribution to the overall care.

The patient with cognitive impairment was an 88-year-old woman living independently in the community near one of her five adult children. She was diagnosed with mild cognitive impairment seven years ago and was prescribed Donepezil (Aricept) delay cognitive impairment. She is also diagnosed with Chronic Obstructive Pulmonary Disease and had been prescribed medication for the same; however, the treatment was discontinued after her condition was stabilized and a further improvement in pulmonary function was not likely. She is also prescribed Levothyroxine at 100 mcg daily, which she takes orally every morning. Prior to the study, she had experienced three episodes of bowel incontinence while outside the home. She had early stages of urine incontinence and was a regular user of pads. She had mobility difficulties because of an accident 30 years ago. Linguistically, she had a working knowledge of English, and some knowledge of French, but was increasingly reliant on Farsi in her conversations and television watching as her impairment progressed. She had independent sources of funds from rental and other businesses and was not under any financial stress.

Her local caregiver was her 67-year-old male child with a history of bladder cancer and asthma. He lived five-minute away. Her distant caregiver was a 69-year-old male child living in a different country and involved in her finances. Both caregivers were still working. The other siblings included a 72-year-old daughter with Alzheimer’s disease, living in a different country; a 66-year-old male child living in a different city, who would provide occasional respite care; and a 60-year-old daughter living in a different city, who was not involved in caregiving.

At the time of enrollment, the local caregiver reported stress and frustration as the sole caregiver. He was responsible for all household activities, including cleaning, meals, and managing his mother's functional and behavioral symptoms. He worked during the day but prepared her breakfast and lunch every morning. He handled management of incontinence issues. He was responsible for medication intake and verified each morning if right medication was taken the night before. The local caregiver was well educated with regard to cognitive impairment disease. The key aspects of burden identified by the local caregiver included frustration with time it took to shop, feed, and maintain the health of the mother, the mother’s mood issues related to social isolation, and increasing difficulties managing disease symptoms, such as incontinence.

When enrolled in the distributed caregiving, the local caregiver daily time burden was reduced from one hour and 45 minutes per day to approximately one hour. This was primarily due to shifting of social conversation from local caregiver to distant caregiver. 

Besides socialization, caregivers also worked together to reinforce several behavioral changes. They encouraged self-grooming through reinforced reminders. The person with cognitive impairment had stopped brushing her teeth and it was helpful when both caregivers would encourage the task. Caregivers also worked together on a method of checking medication intake, placing a single pill by the bed for the evening dose and single pill by the sink for the morning dose, which the caregivers were then able to confirm adherence to via the video link. 

## Discussion

Distributed caregiving was an effective and rapid method of engaging distant caregivers in replacing specific tasks of the local caregiver. The technology was easy to organize and acceptable to caregivers and the elderly person, who eventually learned to use it as a replacement for all her phone calls. In this article, we presented how technology facilitated primarily socialization and entertainment. We are working on the distribution of other caregiving activities including the use of the remotely managed microwave for cooking; the use of timed voiding for bladder training and the use of reminders to guide brushing teeth and grooming. 

Many activities can be automated and simplified so independently living elderly individuals can succeed in completing them. Unfortunately, some activities cannot be automated easily or in a flexible manner that would allow machines to help without supervision. In these circumstances, human-in-the-loop simplification and automation activities might be a reasonable recourse. In the literature, there is extensive discussion of interventions for caregivers of people with cognitive impairment [11-13]. However, full automation requires multicomponent approaches, such as self-maintenance of equipment, which may be beyond the means of current technology. 

Studies that hope to establish the efficacy of innovative assistive technology often focus on designing new products, instead of working with those that are readily commercially available and already accepted for use. Earlier in this paper, we reference several robots developed for artificial conversation. These robots have been shown to be practical and of use to the elderly, but none engage the rest of the family and friends in the care of the patient. Thus, it is not clear if they reduce the caregiving burden. In contrast, the proposed use of home platforms is entirely focused on distributing the burden of care through technology to distant caregivers. The proposed solution is robotics with human-in-the-loop technology. It displaces responsibility for many tasks that robots try to automate to a distant human and therefore offers more flexibility.

To our knowledge, this is the first occasion where distributed caregiving has been described and demonstrated. Most current robotic solutions do not involve other members of the patient’s existing support group and therefore miss an important opportunity to reduce caregiver burden. The technology used in this case study is widely available and relatively inexpensive. This is just one example of how technology can be used to increase the care network and reduce the physical and psychological burden on the primary caregiver at a relatively low cost. However, interventions must be targeted around the specific needs of the case.

Future studies should extend the care to other instrumental daily living activities such as

(a) The use of remote management of kitchen devices to enable elderly persons with cognitive impairment to safely cook for themselves.

(b) More advanced methods to remotely monitor medication intake.

(c) Leveraging timely reminders and remote problem solving to facilitate grooming. Often elderly are looking for specific items to groom, and remote caregiver guidance can help them find what they are looking for.

(d) Platforms to enable both caregiver and self-directed toileting, as instructions have been shown to be effective in reducing incontinence [30-32]

(e) Automation of shopping for food and food delivery.

(f) Reducing the probability of falls in the night by turning lights on at times when elderly individuals are trying to get out of bed.

The full potential of home platforms to reduce the burden of caregiving has not been explored and additional studies are needed to accomplish these tasks. There are clear challenges and limitations associated with distributed caregiving and the use of home platforms. It is inherently reliant on the presence of a distant family member who is willing to engage in care. Often the primary caregiver is also the only available caregiver, even if distant communication with other family members is possible. Furthermore, technology may increase conflict among caregivers. Everyone may not agree on how their loved elderly person should be cared for. Technology increases transparency in the care of the elderly and the increased level of awareness may lead to more family conflict.

## Conclusions

While the impact of distributive caregiving on caregiver burden was assessed in this case study, there was no formal assessment of the impact on the patient’s health outcome. However, there were no obvious negative effects on the patient, and the known increased medication adherence as well as social engagement both have the potential to improve the patient’s outcome significantly. Additional studies are needed to further expand distributed caregiving and evaluate its impact on all parties involved.

The system introduced in this case study is a creative, accessible, low-cost, and sustainable intervention to address the complex and interconnected issues that emerge in reducing the burden of caregiving. A significant challenge for the healthcare system is to reuse existing technologies to make living independently more feasible for people with cognitive impairment. We hope that the broader use of both distributed caregiving and home platforms will prevent institutionalization, and perhaps someday even allow the elderly currently in nursing homes to return to community living.

## Appendices

Appendix A

The following Python code shows how home platforms can be used to enable distant caregivers to change television channels for a person with cognitive impairment.  The Python code was used on a Windows computer within an internal network.  The Python code was obtained from GitHub and was adjusted to suit this case study [33].

The home platform for entertainment consisted of a Smart TV and a Roku device.  The Roku device is essential for streaming media from the internet to the TV.   The Roku device was connected to a Vizio Smart TV and set up prompts for installing Jadoo TV (a channel that offers programming in Farsi) were followed. Scenario B below shows the code for how installation of the Jadoo TV app can occur using Python.

“”” Scenario A: Begin code for controlling live TV channels (i.e. non cable or specialty channels) ”””

from roku import Roku

import time

# This step requires the TV’s IP address
 

roku = Roku('xxx.xxx.xxx.xxx')

# Create functions for actions to be done on the TV
 

def setChannel(ch):

    time.sleep(1)

    roku._post('/keypress/PowerOn')

    time.sleep(1)

    roku['tvinput.dtv'].launch()

    time.sleep(10)

    roku.literal(ch)

def goHome():

    time.sleep(1)

    roku._post('/keypress/Home')

def reverseMedia():

    time.sleep(1)

    roku._post('/keypress/Rev')

def fastForwardMedia():

    time.sleep(1)

    roku._post('/keypress/Fwd')

def playMedia():

    time.sleep(1)

    roku._post('/keypress/Play')

def select():

    time.sleep(1)

    roku._post('/keypress/Select')

def moveLeft():

    time.sleep(1)

    roku._post('/keypress/Left')

def moveRight():

    time.sleep(1)

    roku._post('/keypress/Right')

def moveDown():

    time.sleep(1)

    roku._post('/keypress/Down')

def moveUp():

    time.sleep(1)

    roku._post('/keypress/Up')

def goBack():

    time.sleep(1)

    roku._post('/keypress/Back')

def powerOn():

    time.sleep(1)

    roku._post('/keypress/PowerOn')

def powerOff():

    time.sleep(1)

    roku._post('/keypress/PowerOff')

def channelUp():

    time.sleep(1)

    roku._post('/keypress/ChannelUp')

def channelDown():

    time.sleep(1)

    roku._post('/keypress/ChannelDown')

def volUp():

    time.sleep(1)

    roku._post('/keypress/VolumeUp')

def volDown():

    time.sleep(1)

    roku._post('/keypress/VolumeDown')

def volMute():

    time.sleep(1)

    roku._post('/keypress/VolumeMute')

## Test the code

# Turn on the TV

powerOn()

# Turn the volume up

volUp()

# Select channel 4.1

setChannel("4.1")

# Turn off the TV

powerOff()

“”” Scenario B: Begin code for navigating to branded TV channels. In this example, BBC on Jadoo TV, speaking Farsi language.  You will need all the code created in Scenario A as well.”””

“”” Determine the types of apps available in the Roku.  If the output does not show the desired app, you will have to add it from the Roku Setting.”””

roku.apps

# Show the Jadoo TV Farsi Home Page on your Roku TV

jad = roku[‘Jadoo TV Farsi’]

jad.launch()

# Select LIVE TV from the Jadoo TV Farsi App Home Page

select()

“”” Watch the TV monitor and navigate to the desired language using Python to manipulate selections.  In this case we are selecting the Persian Language”””

# Select the Persian Language from the pull-down menu

select()

moveDown()

select()

“”” Use the Up, Down, Left, Right movements to select BBC News icon. Locations may vary based on your TV and Device”””

for i in range(7):

    moveDown()

# Move right four times to get to BBC News icon

for i in range(4):

    moveRight()

select()
